# Team functioning across different tumour types: Insights from a Swiss cancer center using qualitative and quantitative methods

**DOI:** 10.1002/cnr2.1541

**Published:** 2021-09-28

**Authors:** Felicitas Hitz, Karin Ribi, Gudela Grote, Michaela Kolbe, Christof Schmitz, Benjamin W. Lamb, Thomas Ruhstaller, Peter Berchtold, Nick Sevdalis

**Affiliations:** ^1^ Oncology Haemtology Kantonsspital St.Gallen St.Gallen Switzerland; ^2^ International Breast Cancer Study Group Coordinating Center Bern Switzerland; ^3^ Department of Management, Technology and Economics ETH Zürich Zürich Switzerland; ^4^ Department "Simulationszentrum" University Hospital Zürich Zürich Switzerland; ^5^ College M Bern Switzerland; ^6^ Department of Urology Cambridge University Hospitals NHS Foundation Trust Cambridge UK; ^7^ Faculty of Health, Education, Medicine and Social Care Anglia Ruskin University Cambridge UK; ^8^ Brustzentrum Ostschweiz and University of Basel St.Gallen Switzerland; ^9^ Centre for Implementation Science Health Service and Population Research Department, King's College London UK

**Keywords:** cancer management, behavioral science, cancer care, cancer education

## Abstract

**Background:**

Multidisciplinary care is pivotal in cancer centres and the interaction of all cancer disease specialists in decision making processes is state‐of‐the‐art.

**Aim:**

To describe differences of MDTMs by tumour type.

**Methods:**

Twelve multidisciplinary team meetings (MDTMs) with participation of different cancer disease specialists at a tertiary hospital were assessed by an exploratory sequential mixed method approach with interviews, observations and a survey to address the following five topics: organisational structure and supporting technology; leadership; teamwork; decision‐making, perceived value and motivation. Thirteen persons with different tumour specialities and levels of seniority were interviewed. The 12 MDTMs were observed twice by uninvolved persons and evaluated by the participating physicians with a survey.

**Results:**

There were no systematic differences between MDTMs for different tumour types with the exception of the non‐disease specific type MDTM, which was the only one for which the organisational structure was not driven by an electronic tool. However, several factors could be identified that generally influenced the functioning of the MDTMs. In particular, the quality of decision‐making was highly dependent on the availability of case‐based information and the presence of relevant cancer disease specialists. Leadership and teamwork were rated as important and were comparable across the MDTM. Team participants' motivation and perceived value of MDTMs was high across all meetings.

**Conclusion:**

MDTM at a single institution did not demonstrate disease specific characteristics. An effective MDTM, irrespective of the tumour type, can be successfully structured by technical means and a chairperson coordinating the interaction of cancer disease specialists to improve the decision‐making process.

## 1 BACKGROUND

Multidisciplinary team meetings (MDTMs); otherwise termed tumour boards or cancer conferences) are a key element for care planning by cancer disease specialists at cancer centres internationally.^1^, ^2^ Such teams typically include surgeons, oncologists, radio‐oncologists, pathologists and radiologists (and in some settings cancer nurses) involved in the case‐based discussion of a cancer patient and decision making about their care. The underlying premise of MDTMs is to facilitate cross‐speciality collaboration and to elicit a multidisciplinary expert review of a patient's tumour and condition, which would not be available through a single cancer specialist. This multidisciplinary aspect of cancer care is well recognised and was recently underlined by an initiative of European representatives of cancer disease specialists.^3^ The MDTM is therefore the main vehicle for the elicitation of expert clinical opinions on recommended patient care; and for the integration of these opinions into the decision‐making process for each patient.

As observed in other areas of healthcare delivery,^4^ the task of a MDTM of coordinating several cancer disease specialists within the multiple treatment options for an individual patient is complex and can be a challenge.^5^, ^6^ Cancer treatment processes managed by teams are subject to several internal and external influences.^7^ Collaboration and teamwork are increasingly recognised as central aspects of a successful cancer patient care.^8^ In the wider healthcare literature, the argument has been consistently made over the past decade that team functioning can be improved through analysis of a team, identification of any deficits and implementation of interventions.^9^, ^10^ How to specifically support the way cancer MDTMs function, however, remains less well understood. While there is a plethora of research on decision‐making in organisations and teams^11^ in medical institutions,^2^, ^12^ literature on teamwork and leadership in cancer MDTMs and its implications for patient care is only beginning to emerge.^13^, ^14^ This research has started to demonstrate that factors enabling MDTM decision‐making are defined by both objective parameters and subjective factors. Objective parameters include facets such as availability of radiology and pathology reports and the presence of all required cancer disease specialists at the MDTM. Subjective factors include aspects of team working such as the quality of team members' contributions to case reviews during meetings, and the team leaders' inputs.^15^, ^16^

One of the questions that to‐date remains debatable is whether MDTM‐working is broadly determined by the same human and organisational factors across all tumour types, or in contrast, whether successful MDTM‐working modes are tumour‐specific. Evidence on this is very limited. A national self‐report survey study from the UK analysed data collected in 2009 on MDTM‐working across different tumour types. The study revealed that MDTMs in the UK were largely in agreement regarding their set up.^17^ However, there were some significant cross‐tumour differences in a few areas, including meeting preparation, selection of patients for MDTM review, and the overall perceived usefulness of the team inputs into the decision‐making. Further, this study revealed that haematology team members responded significantly differently compared to solid tumour experts. We are not aware of any further studies addressing specifically this question, which is important from the perspective of infrastructure setup and support offered to cancer teams in large cancer centres globally. This is an important evidence gap, which needs addressing before we consider potential team support or training interventions for cancer teams and MDTMs.

This study aimed to start addressing this gap by evaluating all MDTMs at a cancer centre in Switzerland. Our primary objective was to determine whether the studied MDTMs differed by tumour type regarding their supporting organisational and technological structures, leadership, and a wide range of human factors – including quality of team working, decision making and perceived value of the MDTM to cancer care. Our secondary objective was to add to the evidence base on MDTM organisation and support. In doing so, we aimed to produce evidence to inform Swiss cancer policy, as there is currently no formal guidance regarding how to structure and organise cancer teams in Switzerland.

## 2 METHODS

### 2.1 Study design and setting

This was a descriptive mixed‐method study, which used an exploratory sequential design including a number of data collection techniques to collect qualitative and quantitative data on MDTM‐working.^18^, ^19^ The study was conducted at a single institution of a tertiary hospital in Switzerland. The study was reviewed and approved as a quality of care evaluation by the relevant hospital board; and all participants consented to the study data collection procedures (described below) prior to the data collection.

### 2.2 Study sampling framework and procedure

At the time of study conduct, 12 MDTMs for different tumours were established at the hospital. All of these teams and the staff attending the MDTMs represented the study population and were eligible for inclusion in the study (see section 2.3 for details of the sampling done for the different study phases/methods).

The study was carried out in multiple phases including a characterisation of MDTMs and their practices, exploratory interviews with individual team members, real‐time observations of MDTMs, and a self‐report survey for all MDTM members. To address the primary objective, the types of data collected through the different methods are summarised according to the five main topics of interest (Table 1).

**TABLE 1:** Topics addressed within the interview, observation and survey by qualitative and quantitative methods

		Interview (sample questions)	Observation	Survey
	Topic			
1	Organisational structure and supporting technology	Who is participating? What information is presented and how do you rate the quality of it? Is there a case selection? How do you rate the quality of infrastructure, technical support?	patient history imaging pathology report comorbidity psycho‐social information patient known by the presenting physician	Type of MDTM meeting just attended Participants position (hierarchy levels) Participants specialty
2	Leadership	What constitutes ideal leadership for you? What do you like/dislike about the way the meeting is led? What behaviour do you expect from the leader in case of ambiguous/difficult discussions	Time management Case prioritisation Chairing by enhancing team work and decision making Facilitate the discussion/listening and communicating Ability to summarise cases using information that emerged during MDTM and formulate a decision Keeping meeting focused Management of disruptive personalities and/or conflicts Allowing/encouraging all team members to contribute Creating a good working atmosphere	Prioritisation of cases is adequate Time management and quality of decision is in equilibrium Divergent opinions are included in the discussion Discussion of controversies is avoided Own contribution to patient case discussion is appreciated by leader Decision based on best qualified person, irrespective of hierarchic level
3	Team work	How is the quality of the discussion among meeting participants? How is the atmosphere during the discussions Do you feel that your contribution is appreciated/supported by leader/colleagues?	team contribution	MDTM atmosphere is open to discussion sensitive topics and controversies The way of interaction among participants enables decision‐making Own competencies are asked from colleagues The board is open for criticis The final decision is based on consensus among participants
4	Decision making	Is always a decision taken? Is there a protocol of the decision? What is needed to improve the decision making process?	Decision taken (yes, no, deferred to next MDTM) Each patient discussed has a clear treatment plan	The final decision is based on consensus among participants Decision‐making is based on expertise independent of position or hierarchy of opinion leaders
5	Perceived value and motivation	How do you rate the MDTM regarding its structure and effectiveness' What do you like/dislike about the MDTM? Do you think that MDTM have an educational benefit? Do you have time to participate? What is your role at the MTC meeting Do you think you can perform your role as expected?	Not applicable	One question addressing the perceived value of MDTM with seven response options: better diagnostic and therapeutic decisions coordination of patient care furthers the safety of the therapy securing the influence of my/our discipline exchange of information between participting disciplines continued training of the attendees the observance of the regulations of certification One question addressing the motivation for participation with eight response options: need of diagnostic or therapeutic decision for patient collegiality others benefit of my knowledge time available today help for decision‐making educational benefit substitute for colleague obligation

*Note*: Observation: A 1–5 scale to evaluate was used, being 5 the best answer on the scale with the following interpretation: high (4–5); moderate (2–3); low (0–1). Survey: Rating of 1–7 of a Likert scale was used.

### 2.3 Data collection

#### 2.3.1 MDTM characterisation

The main characteristics of MDTM including organisational structure and procedures in place (e.g., type of IT support, meeting frequency and leadership etc.) were collected by the first author (FH) for each of the hospitals' MDTM based on current daily practice. These aspects of MDTM‐working can impact on the team interactions, hence were collated for a subsequent synthesis of the findings.

#### 2.3.2 Interviews with team‐members

The exploratory interviews aimed at obtaining detailed information on the functioning of the teams from individual MDTM members and to develop a better understanding of the complex social processes and potentially controversial issues around MDTMs in order to inform the content of the quantitative survey. The semi‐structured interviews were based on an existing interview guide used with UK cancer MDTMs and adapted for the purpose of this study.^20^ Key questions addressed participants' opinions on MDTM attendance/role as participant, information presentation, case discussion, leadership, decision‐making process, facilitators/barriers to effective team working, the motivation to participate and the value of the MDTM including their educational benefit (Table 1).

Interviewees were sampled purposefully to ensure representation across different specialties and hierarchy levels. Thirteen interviews with 10 heads of department/consultants and three trainees were conducted face‐to‐face covering the following specialties: pathology (*N* = 1), radio‐oncology (*N* = 1), surgery (*N* = 3), oncology (*N* = 3), radiology (*N* = 1), haematology (*N* = 1), neurology (*N* = 1), dermatology (*N* = 1) and gynaecology (*N* = 1). An interviewer experienced in conducting semi‐structured interviews (KR), who was not a member of any of the MDTMs conducted the interviews. Oral consent was given by the interviewees before the recording started. Each interview lasted between 30 and 45 min; all interviews were audiotaped and transcribed verbatim. Each transcript was analysed by two researchers independently using standard recommended analytic techniques.^21^ In brief, the coding was thematically guided by the interview questions and complemented with an inductive thematic analysis. In addition, one of the researchers (MK) specifically searched for phenomena and processes known from research on decision making in groups,^22^ multi‐disciplinary team^23^ and communication culture in organisations,^24^ which may be implicitly expressed by interview participants. The identified themes were discussed among all authors to decide how they would subsequently be used to inform the content of the study survey. Verbatim quotes were extracted from the interviews to illustrate the identified themes.

#### 2.3.3 Real‐time MDTM observations

For the real‐time team observation during MDTM two assessment tools were applied, the Metric for the Observation of Decision‐making (MDT‐MODe)^20^, ^25^ and the ATLAS tool.^26^ The MDT‐MODe has been developed specifically to assess cancer teams' interactions in real‐time and was validated for use across several tumour types. It allows a quantitative assessment of patient and disease related information discussed during the MDTM (patient history, imaging, pathology, comorbidity, psycho‐social information, patient knowledge by the presenting physician) and also assesses the level of team working during a case review. The validated ATLAS tool has been developed to assess elements of team leadership in the context of cancer care with 12 chairing criteria (Table 1).

We trained 12 observers, six with a clinical and six with a non‐clinical background. All of the observers have never participated in MDTMs before. The tools were translated into German using forward‐backward translation. The training included explanation of the tools content and how to apply them supported by illustrative videos of good and poor teamwork behaviours in cancer MDTMs. Each MDTM was subsequently observed once by a pair of observers (one with, one without clinical background) who were present during the meeting. The different criteria included in the MDT‐MODe and ATLAS were rated based on verbal descriptions of behaviours on three levels corresponding to 1 = poor, 3 = average, and 5 = excellent.

Observations were analysed descriptively (means and standard deviations) for each criterion and according to MDTM. If available, we first calculated the mean of the two observers for each patient. If one observer had been unable to provide a rating for a specific element included in MDT‐MODe or ATLAS, we included the single rating in the analysis. Means and standard deviations were calculated for each element scored. We also descriptively analysed the MDTM treatment decision taken for each patient (decision taken; decision suspended; or observers did not agree).

#### 2.3.4 Team‐members survey

The self‐report MDTM‐members' survey was developed based on the themes identified in the interviews (Table 1). The survey included items addressing teamwork (six items), leadership (five items) and decision‐making (two items) in MDTMs. Participants were asked to rate if they agree with each statement on a scale ranging from 1 = not at all to 7 = absolutely. We further assessed motivation for participation in the meeting (single item, with eight response options) and perceived value of the MDTM a participant had just attended (single item, with seven response options). The questionnaire was pilot tested with regard to feasibility and comprehension prior to data collection. Data collection took place once at the end of each meeting. Each survey question was analysed individually, with responses to each question summarised descriptively (means and standard deviations) and according to MDTM. There was no formal statistical testing of differences observed between MDTMs – as we did not have any such hypothesis to formally evaluate. The study was therefore not designed or powered to evaluate cross‐MDTMs differences.

In the final stage of the study, all data from the different sources were reviewed synthesised in order to produce a global assessment of MDTM working across tumour types.

## 3 RESULTS

### 3.1 Characteristics of MDTM's

Table 2 gives an overview of the 12 assessed MDTMs with respect to the meeting frequency, use of an electronic database, team leadership/chairing, focus of decision‐making, average number of attendees and their specialties. The MDTMs at the study site typically worked as follows: registration of patients to be reviewed was carried out in advance. Patient case registration was based on a common electronic database, to which all physicians had access. The database structured the patient case information for the stage of the tumour and the information needed from the MDTM members. At the meetings, patient cases were presented by the treating physicians, followed by a review of case‐based imaging and pathology reports. The decisions were based on clinical/scientific considerations and were not designed to include patients' preferences at the time of the MDTM – though these preferences were expected to be taken into account when the patient saw his/her treating physician subsequently to the MDTM. MDTM attendees and decisions for all reviewed patient cases were recorded. Case discussions were focused on both diagnostic and therapeutic decisions within the same MDTM, except for the breast MDTM, which was divided in two meetings: a diagnostic (pre‐operative) and a therapeutic (post‐operative) MDTM. The 12 MDTMs usually lasted between 30 and 45 min and were attended by a total of 183 physicians of different specialties involved in cancer care with different levels of seniority (Table 2). Cancer nurses or patients did not attend the MDTMs.

**TABLE 2:** Characteristics of studied MDTMs and participants

	Multidisciplinary teams by tumour type	Frequency	E‐register	Meeting leader/chair	Focus of decision‐making	Average number of team‐members present per MDTM	Cancer disease specialists in attendance
1	Gastrointestinal malignancy	Twice weekly	Present	explicit	Therapeutic > diagnostic	20	a
2	Thoracic malignancy	weekly	Present	explicit	Therapeutic > diagnostic	15	a
3	Central nervous system malignancy	weekly	Present	implicit	Therapeutic > diagnostic	6	b
4	Urologic malignancy	weekly	Present	implicit	Therapeutic > diagnostic	8	b
5	Haematology	weekly	Present	explicit	**Diagnostic>** **therapeutic**	10	b
6	Non‐disease specific board	weekly	Absent	implicit	Therapeutic > diagnostic	10	c
7	Breast pre‐operative	weekly	Present	explicit	**Diagnostic >** **Therapeutic**	10	a
8	Breast post‐operative	weekly	Present	explicit	Therapeutic > diagnostic	10	a
9	Gynaecologic malignancy	weekly	Present	explicit	Therapeutic > diagnostic	15	a
10	Ear‐nose‐throat malignancy	weekly	Present	implicit	Therapeutic > diagnostic	20	a
11	Soft tissue and bone malignancy	Fortnightly	Present	implicit	Therapeutic > diagnostic	10	a
12	Dermatologic malignancy	monthly	Present	implicit	Therapeutic > diagnostic	6	b

*Note*: a – participation of surgeon, medical oncologist, radio‐oncologist, radiologist, pathologist. b – participation of surgeon, medical oncologist, disease related specialist as neurologist, dermatologist, haematologist. c – participation of surgeon, medical oncologist; no presence of radiologist, no pathologist.

Some differences between the setup of MDTMs were also identified. The breast, gynaecological and gastrointestinal MDTMs had a predefined chairperson who led the team through the discussion of the different cases and predefined standard procedures for the typically presenting cases. These three MDTMs also included participants from external collaborating hospitals by real‐time via videoconference. For the remaining MDTMs, there was no clearly identified chairperson to lead the discussion. Further, the single non‐disease specific MDTM had no patient registration in advance of the meeting.

### 3.2 Exploratory interviews with team‐members

Individual interviews revealed that participants of MDTMs were prepared for their case presentation. The standardisation of information presentation and the prioritisation of cases based on their complexity were factors seen as contributing to the efficiency of MDTMs:

*The diagnosis is shown, the underlying disease, secondary diseases. Histology, images, ‐ X‐rays, CT images, MRI's are presented. Everything that is done in terms of examination and diagnostics for this case is discussed together and interactively (I13, head of department)*

Interview participants considered the chairperson's ability to structure the discussion, prioritise cases, control time management, value individual opinions and leading through controversial discussions as important attributes of effective team leadership:

*Someone who bundles all the information that is being discussed and then makes the final decision and says: This is how we're going to do it now. But everyone has to agree (I3 trainee)*.

They also considered the expertise of each participant an important feature to successful MDTMs, but indicated that the meeting should not be dominated by opinionated individuals:

*The tumour board is only as good as the individuals who are on it. The more experience they have, the better the professional quality of the decisions (I2, head of department)*.

Problematic issues in MDTMs included hidden conflicts between chairperson and disease experts, unprepared experts, and case presentations that were inserted into the meeting late (hence lack of preparation for them) or had missing information.

Interview participants mentioned the ability to reach consensus, good camaraderie within the team, a psychologically safe atmosphere and a constructive culture of debate as facilitators for effective team working:

*One is allowed to speak freely and is not somehow cut off or 'Be quiet now'. Yes, it is actually a discussion with each other. At least in this tumour board it is a ‘with each other’ (I3 trainee)*.

In contrast, barriers identified by the interview participants included power struggles between specialities, the imposition of clinical opinions by individuals, different views on who is qualified to take a decision and decision‐making occurring outside of the MDTM:

*The different points of view are problematic, or: Who has sovereignty over patients? The surgeon thinks differently than the oncologist or the gastroenterologist. And sometimes narcissistic personality structures come into play, which then make the decision no longer objective (I6, Head of department)*.

All interview participants shared the opinion that the attendance of cancer disease specialists from different disciplines is necessary for successful decision‐making, but they also indicated that it might be a platform for demonstrating individual power:

*Now and then because of political infighting ‐ so to speak – the meetings take a bit long. It becomes more of a power show than really patient‐centered (I7, Head of department)*.

Lastly, MDTMs were seen as a forum for clinical education. However, the motivation to participate was reported to be sometimes hampered by lack of time or conflicting clinical priorities:

*The potential for education is very high, but there are far too few trainees attending the meetings (I8, Head of department)*.

### 3.3 External observers' ratings of MDTM

The observer analysed 153 patient cases, with a minimum of four and a maximum of 36 patient cases per MDTM and 8–72 observations, respectively. Ratings from both observers were available for 137 of these patient cases. MDT‐MODe was the tool used to score patient discussions.

The ratings for patient and disease related information regarding patient history and patient being known by the presenting physician resulted in high mean scores. Imaging was always present, whereas pathology reports were presented with large variation across the MDTMs. The discussion of psycho‐social information and patient's preferences were scored low in all of the observed meetings (Table 3).

**TABLE 3:** Observer ratings for patient and disease related information in means and standard deviation

Type of information	Decision taken (*n* = 125)	Decision suspended (*n* = 10)	Observers did not agree (*n* = 18)
Patient history	4.8 ± 0.6	4.2 ± 0.9	4.8 ± 0.5
Imaging	3.8 ± 1.7	2.8 ± 2.0	2.8 ± 1.9
Pathology	2.4 ± 1.3	2.5 ± 1.2	2.2 ± 1.0
Psycho‐social information	1.6 ± 1.0	1.9 ± 1.2	1.5 ± 1.1
Comorbidity	2.1 ± 1.3	1.4 ± 1.0	1.2 ± 0.6
Patient's views represented by the physician	1.6 ± 1.1	1.0 ± 0.0	1.4 ± 0.8
Patient seen by the presenting physician	4.7 ± 0.5	4.7 ± 0.8	4.5 ± 0.8

The observations of experts' contribution from and radiology revealed largely divergent results for different MDTMs. Most striking was the result for soft tissue and sarcoma MDTM with excellent scores (mean ratings above three) for the contribution of radiologists and pathologists. Other MDTMs with presence of these disciplines scored lower (mean rating 1.5 for gastrointestinal neoplasm MDTM) but reached a mean score above three for radiologists.

In 125 (82%) patient cases in the observed MDTMs a clinical treatment decision was reached. Suspended decision (unclear or missing) were reported in 10 cases (7%). Six out of 10 cases (7%) with suspended decisions were in the non‐disease‐specific MDTM. In 119 of 137 cases with two observations the observers were in agreement on whether a decision was taken or not (Tables 3 and 4).

**TABLE 4:** Agreement between observers* regarding the decision taken

	Multidisciplinary teams by tumour type	Decision taken (*n*)	Decision suspended (*n*)	Observers did not agree (*n*)	Total (*n*)
1	Gastrointestinal malignancy	33	0	3	36
2	Thoracic malignancy	12	0	2	14
3	Central nervous system malignancy	8	1	1	10
4	Urologic malignancy	11	0	0	11
5	Haematology	4	1	1	6
6	Non‐disease specific board	10	6	0	16
7	Breast pre‐operative	4	0	0	4
8	Breast post‐operative	5	0	0	5
9	Gynaecologic malignancy	14	0	6	20
10	Ear‐nose‐throat malignancy	10	2	3	15
11	Soft tissue and bone malignancy	8	0	2	10
12	Dermatologic malignancy	6	0	0	6
	Total	125	10	18	153

The observers also used ATLAS to score leadership in the observed MDTMs. The total score for leadership based on the mean rating of the predefined nine categories of the validated observation tool (ATLAS) was good (mean scores 4–5) in five MDTMs and moderate (mean scores 2–3.9) in the remaining seven MDTMs. Prioritisation of complex and urgent patient cases was scored overall low across MDTMs (mean scores 0–1.9).

#### 3.3.1 Survey results

Of the available population of 183 team‐members in attendance of the meetings, we received 181 surveys (response rate = 99%).

The survey participation reflects a typical constitution of MDTM: Seniority levels were represented with 8% heads of department, 32% senior consultants, 30% junior consultants and 30% trainee physicians. Participants' specialties were as follows: 40 medical oncologists, 79 surgeons, 10 radio‐oncologists, nine gastroenterologists, four haematologists, three neurologists, and two dermatologists, laboratory experts included nine radiologists, and 10 pathologists.

**FIGURE 1:**
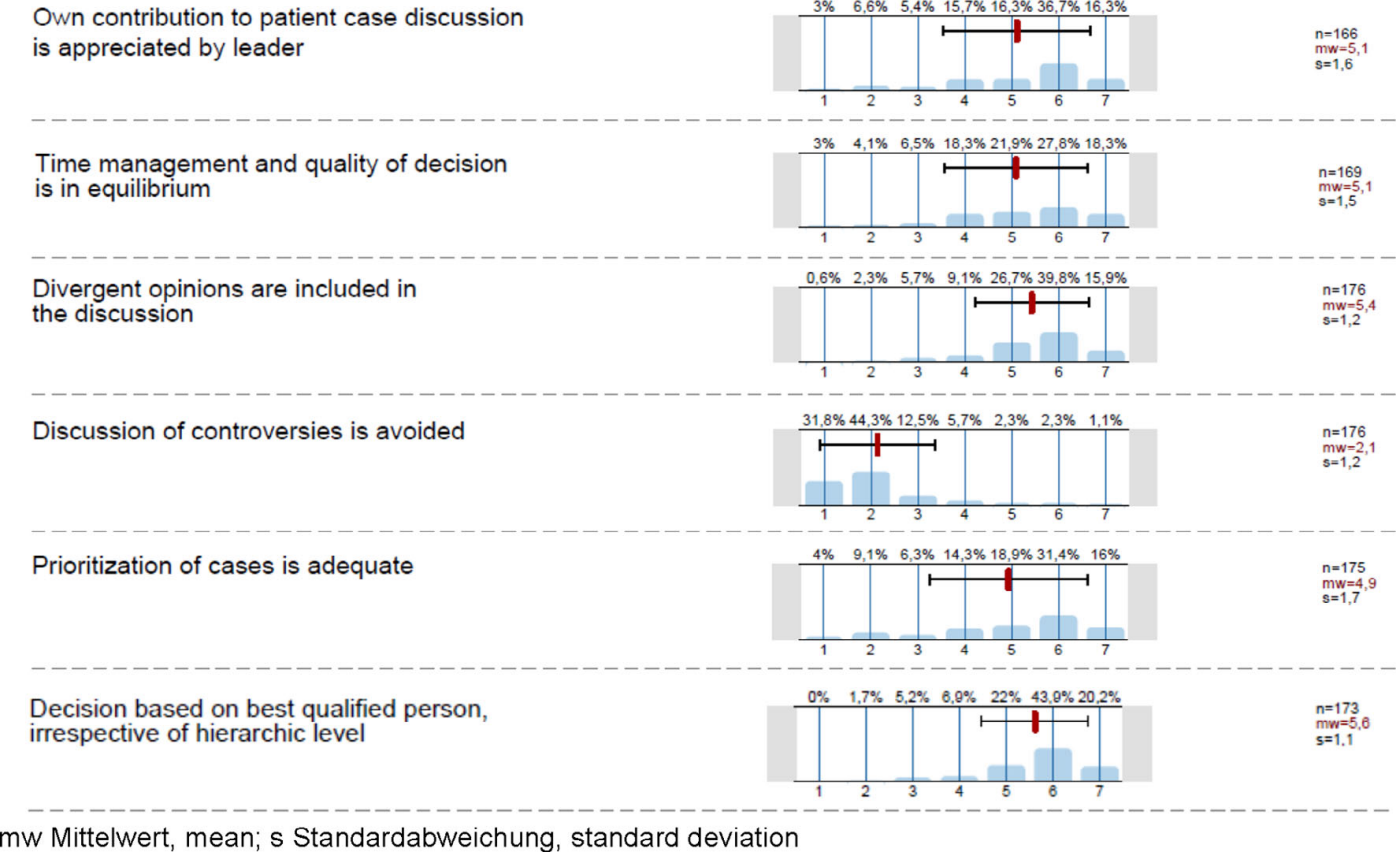
Survey leadership issues

**FIGURE 2:**
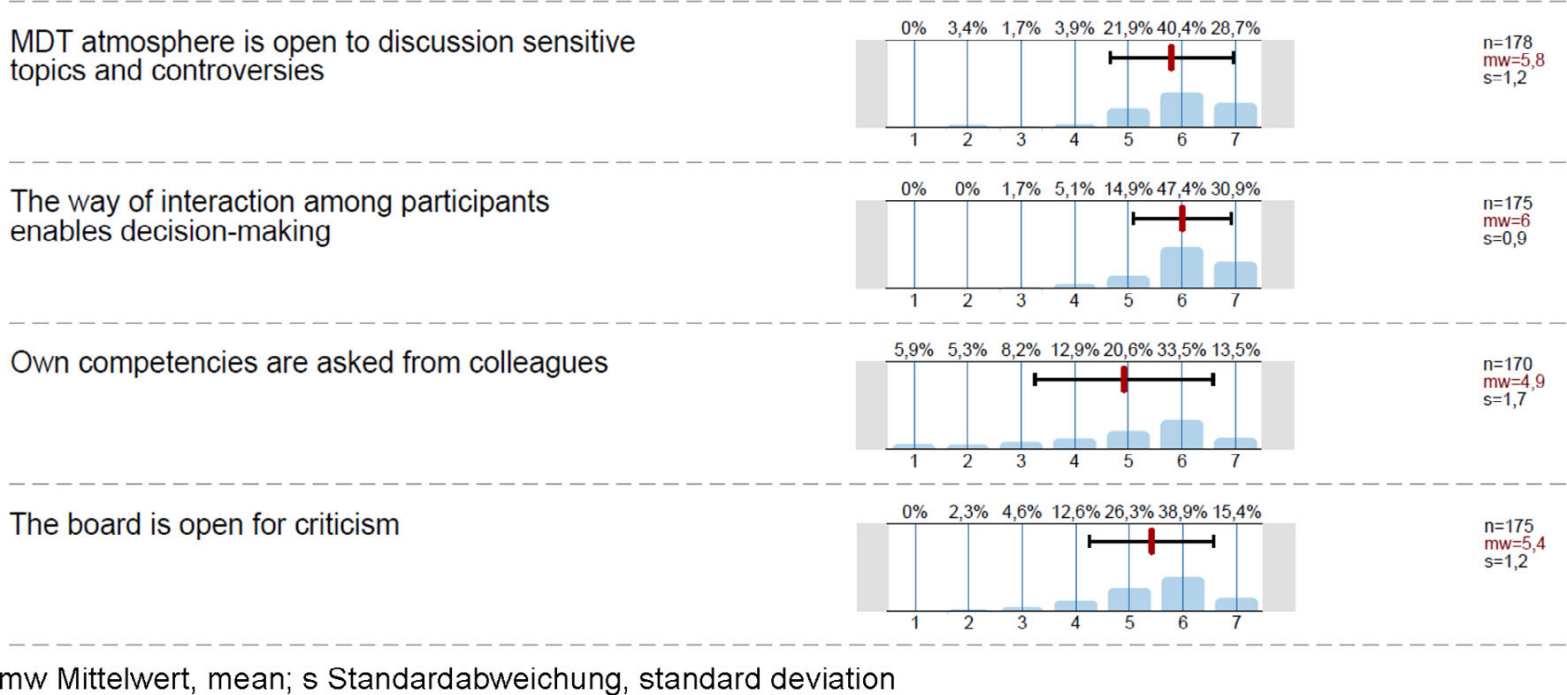
Survey teamwork competencies

Several of the leadership facets mentioned in the interviews were rated quite positively by the MDTM participants (Figure 1). Case *prioritisation* (mean 4.9; SD 1.7) and *time management* received high ratings (mean 5.1; SD 1.6), both showing scores above four. Similar high ratings were given to items focused on *divergent opinions are included in the discussion* (mean 5.4; SD 1.2) *and decisions based on the most qualified person irrespective of the hierarchic position* (mean 5.6; SD 1.1). These results were similar across MDTMs. Only in the MDTM for pre‐operative breast cancer that is exclusively diagnostic the ratings for time management and prioritisation were higher (mean 6.1; SD 0.9). The four questions in the survey that addressed overall teamwork competencies were rated on average between 4.9 and maximal 6.0 (range 1–7 Likert scale) (Figure 2). We were not able to detect meaningful differences of teamwork ratings between the diagnostic (pre‐operative) and therapeutic (post‐operative) breast MDTM. The survey responses indicated a considerable uniformity across all MDTM with respect to decision making: *final decision is based on consensus among participants* (*n* = 177; mean value 5.4, SD 1.3); *decision‐making is based on expertise independent of position or hierarchy of opinion leaders* (*n* = 173, mean value 5.6, SD 1.1) and *the way of interaction among participants enables decision‐making* (mean 6.0, SD 0.9).

**FIGURE 3:**
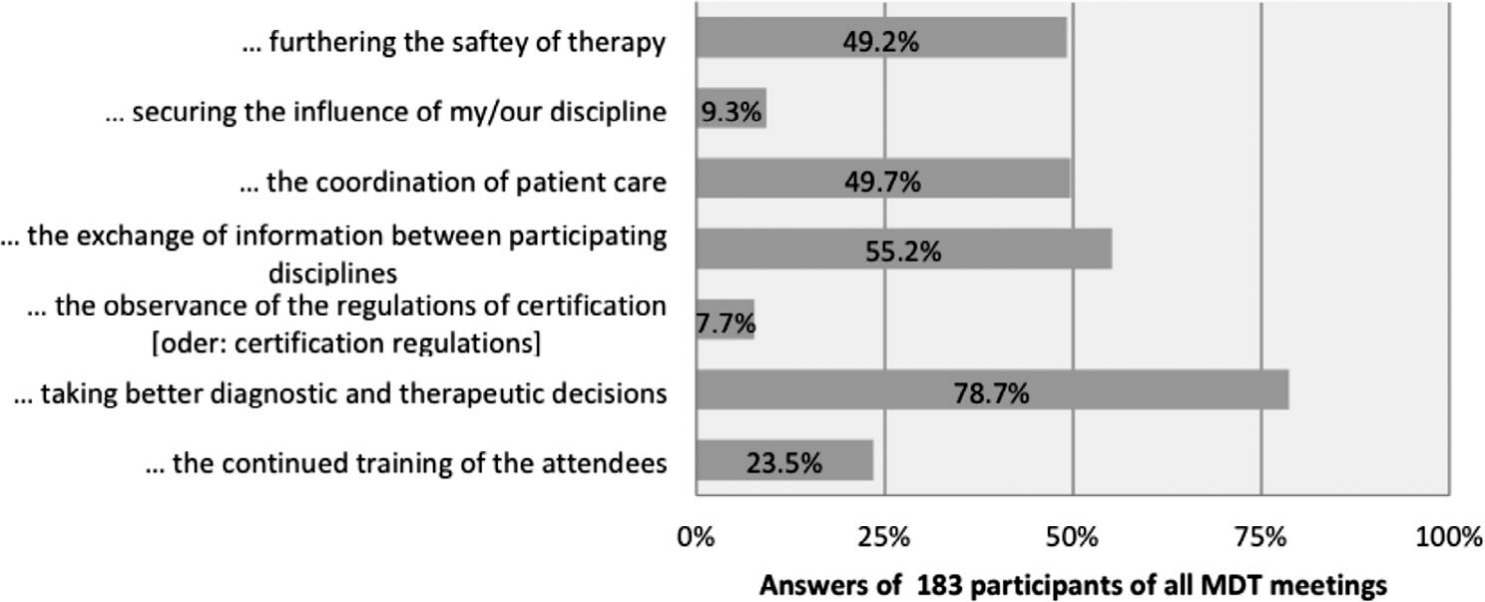
Survey perceived value of all MDTM

Figure 3 displays the perceived value of MDTM: *Taking better diagnostic and therapeutic decisions*, was valued by the participants as the most important contribution of an MDTM. This priority was also seen comparing the disease‐specific MDTMs, the expertise levels and specialities. However, there were some differences seen between experts for several responses: Medical oncologists chose *taking better diagnostic and therapeutic decision* more often than surgeons (*n* = 40; 87% vs. *n* = 79; 76%). Likewise, *coordination of patient care* was also more often chosen by medical oncologists than surgeons (*n* = 40; 62.5% vs. *n* = 79; 47%).

The question addressing the motivation for participation was answered by only 40/183 participants. The most obvious motivation for participation was the need of a diagnostic or therapeutic decision (30%), followed by a commitment to sustain the decision‐making process with personal knowledge and expertise (25%). Motivations appeared to differ across MDTMs. The most striking difference was seen for the visceral surgery MDTM with a much higher number of responses for a commitment to bring in expertise (50%); whereas in postoperative breast tumour the motivation for a diagnostic or therapeutic decision was highest (66%). Across levels of expertise, trainees stated they were typically attending a meeting with the aim to reach a treatment decision by the team, whereas this was least mentioned by senior heads of departments.

Lastly, an educational benefit was expressed overall by 17.5% of the participants. Analysis based on level of expertise/hierarchy indicated that trainees (39%) valued educational benefits higher than junior consultants (20%). Heads of department and senior consultants reported no educational value of the meetings.

### 3.4 Discussion

Our study met both of its objectives. Using multiple methods and sources of data, we were able to characterise in detail the structure and functioning of all MDTMs within the study hospital – thereby offering the first such detailed description of a cancer centre within the Swiss cancer care system. We identified areas where the participants who attend these meetings agree on what ‘works’ and also on where problems may arise. We also established areas where clinical information needed by the teams reviewing the patients; infrastructure support; and team procedures are available and facilitate the decision‐making process^27^; and other areas that seem to be lacking. Lastly, we were able to produce a detailed analysis for a Swiss cancer centre that is directly comparable on how cancer teams work and how they can be supported. Overall, we studied descriptively 12 MDTMs within a single Swiss cancer centre and did not observe obvious differences in the organisational and team working facets across them. Our findings are largely in line with the UK study that surveyed MDTM members across different tumour types and found a largely homogeneous pattern of responses with regards to their set up and team functioning.^17^ From the perspective of cancer care and workforce planning, this is a noteworthy finding. It suggests that policies can largely be applied across MDTMs – provided they address effectively the key parameters of MDTM working within the specific context, as captured for the Swiss context in this study.

In the most recent systematic review of team working in cancer teams that we are aware of, Horlait et al.^8^ reviewed 49 studies carried out across 12 countries. The review offers a framework for considering the evidence base on team working in MDTMs – which includes structural characteristics (of MDTMs, hospitals and healthcare systems), leadership, participation and involvement, and organisational culture. Of these, our study offers insights into the first three. We apply the framework outlined by this review to organise our interpretation of our findings and recommendations.

First on structural characteristics, our study demonstrates the overall beneficial role of clear and supportive structures for the MDTM. The electronic registry of patients was used by all teams, except one and was found to contribute to a straightforward case discussion within an MDTM irrespective of the disease. Electronic registration tools can support and improve the organisation of MDTMs, which appeared to also be the case in our study.^28^ The further potential of clinical decision support technology in MDTMs for assistance, preparation, data collection and documentation of decision is well documented.^29^ Further to the technology, we also found that some MDTMs had an established and clearly defined chairperson – which again was seen as facilitative of the overall case review and an active coordinator of the meeting flow. In light of these findings it follows that IT support is part of the infrastructure host a hospital should provide for the cancer teams. Also, within the teams, a designated chairperson is needed.

Second on leadership, we observed that who chaired the MDTMs was typically defined by the hierarchy of the experts in attendance, such that chairing was typically undertaken by the moist senior person present. Anecdotally, this is not an uncommon practice (in healthcare meetings overall, we believe). In this study, the views of MDTM members in the survey were very positive, and this pattern was confirmed by high scores on the observational assessment of leadership during the meetings. The emerging picture is that, in the centre we studied, leadership of MDTMs seemed to be well‐applied and received. We are unable to comment on whether an alternative model to team leadership might work better, as such a comparison was not available. A recent study revealed that, in naturally occurring cancer MDTMs, in which experts took turns to speak during the meeting, the pace of speech was very fast, such that the natural pauses (i.e., opportunities to speak) were few.^30^ The risk this presents for a fast‐paced MDTM is that the conversation might be dominated by some vocal individuals – a risk that we noted in our interview analyses in this study too. These findings suggest that the role of the chairperson in ensuring the meeting is truly one of multiple voices is critical – and this role might not depend on clinical seniority. We hypothesise here the potential for a MDTM chairing ‘model’ with a meeting chair selected for their chairing skills and even perhaps without direct involvement in the care of the reviewed patients, who concentrates on the process of sharing information and making decisions.^31^, ^32^ Chairs of MDTMs can focus on facilitating case reviews and involve experts into the discussion to ensure their contribution to the decision‐making process.^33^ We would propose to our colleagues who regularly attend (and chair) MDTMs to consider and trial this alternative strategy, and report their experiences with it. Added to this, the evidence suggests that professional development and support to MDTM current chairs should be offered by cancer centers to enable them to undertake their role effectively.

Third, on participation and involvement, we obtained an interesting pattern of findings. One observation of note is the systematic under‐representation at the MDTMs of information about the preferences or comorbidities of the patients under review. This despite the fact that in the majority of the cases there was a person at the meeting who had personal knowledge of the patient. This finding suggests that the teams tended to focus on oncological aspects of the tumour, with less attention paid to the rest of the clinical and psychosocial aspects of care.^34^ The fact that cancer nurses did not attend the MDTMs at that time (they still do not do so in Switzerland) might offer some explanation for the latter part of the observation. Even in healthcare systems where nurses do attend the meetings, such as in the UK, the same lower priority to these facets of care has been shown in observational studies.^35^ We believe that this is an area of potential improvement. Care planning and delivery subsequent to the MDTM can only be enhanced if the full clinical picture is known about a patient, so that the MDTM does not waste time considering options that are clinically not applicable. This can be done using a simple checklist, prepared ahead of the MDTM.^20^ Further, pathway analyses are required to establish at what point of the pathway it is optimal to discuss with the patient their circumstances and preferences. It could for example be that the optimal time to do so is after the MDTMs has arrived at a set of potential recommendations, which will then be communicated by the treating physician.^36^ A second observation in relation to participation and engagement, is the differential motivations across team‐members for attendance and some tensions we discovered. Improved decision‐making and care coordination were more strongly prioritised facets of the MDTMs attendance by oncologists compared to surgeons. This opens up some questions regarding what might add more value to these meetings from the surgical perspective.^37^ Interestingly, trainees in attendance prioritised having a clinical decision to implement at the end of the meeting; and educational objectives were prioritised by overall more junior staff‐members. The integration of teaching elements into tumour MDTMs is reported in some research, though not well documented.^38^, ^39^ The value of a MDTM could be increased by explicitly incorporating continuous medical education (incl. in available clinical trials) and promoting multidisciplinary learning even for experienced attendees.^40^ More studies are needed in how educational approaches can be optimised within cancer MDTMs.

### 3.5 Strengths and limitations

The study has several strengths. We chose to combine qualitative and quantitative methods to reflect both an external view on an MDTM (MDTMs characterisation and observation methods) and an internal view with information coming directly from team‐members (interview and survey methods). We applied validated observational tools (MDT‐MODe and ATLAS) specifically designed for MDTMs and applied by trained observers, which overall worked well in the Swiss clinical setting. The joint application of these methods and analysis of the findings allowed us to produce a comprehensive picture of the studied MDTMs.

Limitations included that the study was carried out at a single institution, hence we cannot be certain to what extent the findings reflect an institutional perspective. The generalizability of our observations to other Swiss cancer centres or elsewhere remains to be tested through multi‐institutional studies. Moreover, due to the purely descriptive nature of the study, we are not able to comment on how well the MDTMs studied performed in relation to an external performance benchmark. Furthermore, we took a sampling approach that maximised breadth, which means we analysed a wide range of cases and numbers of participants across the different MDTMs; this limits our ability for cross‐group or tumour‐specific statistical comparisons due to small subgroup samples sizes. Methodologically, we report findings descriptively as the study was not designed, or powered, to evaluate specific inter‐group differences. We did not carry out member‐checking as part of the interview analysis or a peer‐debriefing as part of the observational data analysis. Lastly, the cross‐sectional nature of the study at a single time point meant a limited sampling window, so we cannot account for the contribution to team functioning of different leaders, experts and caseloads over time. The final results of the MDTM study will be presented to the participants for further development and improvement of the structure and team interaction.

### 3.6 Future directions

To our knowledge this is the first descriptive study addressing in depth the issue of MDTM working practices across numerous tumour types and the first study of this types in Switzerland. Overall, we found a coherent pattern, suggesting that policies and guidelines to support MDT functioning sufficiently address the needs and ways of working of all types of MDTMs in Swiss cancer centres. This study is largely replicating a pattern found in MDTMs globally.^8^ From a research perspective the tools that this study applied, can be used to carry out comparative analyses between cancer centres in Switzerland and beyond.^41^ From a practice perspective, the tools used to characterise MDTMs may also be useful for cancer teams to analyse their own work patterns and identify opportunities for improvement – for example, through the formal appointment of a skilled MDTM chairperson to facilitate the case reviews. The analysis of the MDTM's that we have carried out can be fed directly into practical recommendations for MDTMs to implement locally. We have articulated some of these already – including a defined chairperson, and consideration of allocating the role without reference to clinical seniority. A simple checklist can be used to work up cases prior to MDTM presentation and hence improve the information coverage of clinical comorbidities. From a hospital perspective, offering consistent IT support and professional training in meeting chairing and leadership skills are areas the hospitals should invest in to support their cancer teams. Lastly, further consideration should be given to information about the patient's circumstances and wishes – understanding of the flow of the cancer pathway is needed to identify where this can be done best. We hope that the study offers a baseline for both discussions with health policy makers about advancing the delivery of cancer care in Switzerland and for improvements to be considered to improve routine daily practice in cancer care.^42^

## CONFLICT OF INTEREST

NS is the director of the London Safety and Training Solutions Ltd, which offers training in patient safety, implementation solutions and human factors to healthcare organisations and the pharmaceutical industry. The other authors have no conflicts of interest to declare.

## AUTHOR CONTRIBUTIONS

All authors had full access to the data in the study and take responsibility for the integrity of the data and the accuracy of the data analysis. Conceptualization, F.H., K.R., G.G., M.K., C.S., B.W.L., T.R., P.B., N.S.; Methodology, F.H., K.R., G.G., M.K., C.S., B.W.L., T.R., P.B., N.S.; Investigation, F.H., K.R.; Formal Analysis, F.H., K.R., G.G., M.K., C.S., B.W.L., T.R., P.B., N.S.; Resources, F.H.; Writing‐Original Draft, F.H., K.R., G.G., M.K., C.S., B.W.L., T.R., P.B., N.S.; Writing‐Review & Editing, F.H., K.R., N.S.; Visualization, F.H.; Supervision, F.H.; Funding Acquisition, F.H.

## ETHICAL STATEMENT

The study was performed as a quality assurance project and institutional ethical approval was not required. No patient consent required for this study – no patients involved.

## Data Availability

Data available on request from the authors.
